# Abscess of the Jaws, and Its Treatment

**Published:** 1846-12

**Authors:** S. P. Hullihen

**Affiliations:** Wheeling, Va.


### ARTICLE III.

*Abscess of the Jaws, and its Treatment.* By Dr. S. P. HULLIHEN, of Wheeling, Va.

THIS very common and very painful affection of the jaws, may take place at any period from infancy to old age, but occurs most frequently from childhood to middle life, and much oftener in the upper jaw than the lower. It never attacks jaws where there are no teeth, nor roots of teeth, nor where the teeth are not in some way implicated. It mostly appears in that region of the jaws where the roots of the teeth terminate, and generally requires from two to four days for its complete formation. It is most formidable when it happens in connection with the cuspidati of the upper jaw, or the dentes sapientiæ of the lower, at all other times it appears to be more or less severe in proportion only to the thickness of the alveolus, when it originates in such a manner as to be influenced by that cause. The face always becomes considerably swollen from an attack of this disease, and occasionally the swelling extends to the eye-lid, sometimes to the neck, and sometimes to the tonsil of the affected side, and the jaws not unfrequently become entirely closed for a time from the same cause. There is always more or less disturbance of the general system accompanying this complaint, which should be regarded as the effects of the disease, and not as the exciting cause.

Abscess of the jaws originates most frequently within the internal cavity of a tooth; it may, however, occasionally take place from mechanical violence within the alveolus; and still more rarely from a conjoined inflammation of the gum, the periosteum of a fang, and of the alveolus. All cases that may occur from other causes, if not entirely unique in themselves, are decidedly anomalous in their character, and should not be regarded as having any relation to the common abscess of the jaws save in their location and general appearance.

Up to the time that Thomas Bell published his excellent work on the teeth, this disease was generally designated by the name of "gum-boil." "The very name," says Mr. Bell, "is at once a proof of the mistaken notion commonly entertained respecting its nature, and the means of perpetuating the error. The gum is in fact only secondarily affected, the cause being invariably seated within the alveolus." He therefore proposed, "to call it alveolar abscess as more correctly designating its true nature and situation."* A term which was at once adopted by the profes-

* Bell on the Teeth, page 215.

sion and has been employed ever since—a term, which, on account of its supposed appropriativeness has caused a greater and a more general mistaken notion respecting the true cause and origin of the disease, than ever the use of the term gum-boil could have possibly induced. That this disease does not invariably originate within the alveolus as stated by Mr. Bell, but most frequently within the internal cavity of a tooth, is a fact that can be fully sustained beyond doubt or cavil. That abscess may now and then originate within the alveolus cannot be denied; that it sometimes originates in the gum can be as clearly shown. But cases originating in such situations are extremely rare when compared with those in the internal cavity of a tooth. I will now endeavor to demonstrate. For the purpose, however, of avoiding the total unfitness of the name gum-boil, and the erroneous impression the term alveolar abscess inculcates, I propose to treat of the disease, under the general head of abscess of the jaws, not intending by the name to indicate anything in relation to the origin of the disease, but simply the usual place of its development. As to the origin of the disease the several causes will be separately examined, and the difference in the progress of the disease arising from each cause, will be carefully noticed in the following order:

*Firstly.*—From the suppuration of the dental nerve, and the consequent discharge from the internal cavity of a tooth.

*Secondly.*—From mechanical violence within the alveolus.

*Thirdly.*—From a conjoined inflammation of the gum, and the alveolo-dental membranes.

In submitting the proposition that abscess of the jaws most frequently originate in the suppuration of a nerve, and the consequent discharge from the internal cavity of a tooth, I wish it to be clearly and explicitly understood, that such a result is only claimed in cases where the discharge takes place through the foramen at the apex of a fang, and in no case where it can escape through an opening in the crown or body of a tooth—and that all the symptoms hereafter noticed as being attendant upon this form of abscess, have a strict reference only to where a discharge occurs through the extremity of a fang.

The suppuration of the dental nerve is one of the most common affections to which the teeth are liable, and is most frequently induced from the irritation to which the nerve is so constantly exposed, and in such a great multiplicity of ways through the encroachment of dental caries. It sometimes follows extensive fractures of the teeth, sometimes their partial dislocation, and occasionally, great and sudden exposure of their roots from the receding of the gums and alveolar process. From whatever cause suppuration of the nerve may arise, the symptoms are substantially the same ; the amount of irritation and situation of the tooth governing, however, to some extent the rapidity of its course, and the intensity of the accompanying pain.

The symptoms arising from the suppuration of the nerve are generally very uniform and well marked in their character ; at first a tooth becomes painful only while hot or cold fluids pass over it ; soon, however, the pain will continue for a few seconds after the occurrence of such an irritation; then the paroxysms will be prolonged to a few minutes, and then to a continuous dull heavy pain, the tooth will now appear a little sore, loose, and longer than the rest. These symptoms gradually increase, and with them the intensity of the pain. The gum then becomes more or less tender opposite the end of the root. The pain will begin to dart along the courses of the nerves to the teeth of both jaws, the ear, and temple of the side affected ; the offending tooth will now become exquisitely tender, the face to swell, and a most distressing pain of a throbbing character will be experienced in the jaw until the abscess is formed and its contents begin to be discharged.

When a tooth becomes painful in the manner just described, it is a certain indication that the nerve is about to suppurate. When a tooth is very sore, loose, and longer than the rest ; suppuration of the nerve has then already taken place. When the face begins to swell, and the pain is of a throbbing character, matter is then oozing through the foramen at the apex of the root, and a small sac is forming between the end of the root and its periosteum,* in a short time this sac gives way, and the dis-

* I perceive that Dr. James Robinson, of London, has fully sanctioned my views respecting the suppuration of the dental nerve, which I first incidently offered in an article on tooth-ache, that was published in vol. 1, page charge is effused around, and between the point of the root, and the bottom of the alveolus; the matter is now rapidly secreted, and sooner or later finds its way through the thinner plate of the alveolus, which is most generally the external, and here it is again effused between the soft parts, and the alveolar process, giving rise to all the symptoms and appearances before described as being attendant on the latter stage of abscess of the jaws.

Now and then a case may occur where the nerve suppurates, and the face swells, and both pain and swelling subside without the development of an abscess; in such cases the little sac that forms on the end of the root, does not break as usual during the first few hours of its existence, but continues to enlarge until it becomes the size of a pea, and sometimes larger, and very often remaining in this condition for several months; sometimes the nerve may suppurate without causing the formation of a sac, or any swelling of the face. In such cases the foramen of the roots, probably is too small, or the matter too thick to admit of its escape from the dental cavity; sometimes the nerve suppurates, and the face swells, and a discharge follows, which however escapes from the place of its formation along the side of the root, instead of passing through the alveolus and gum. But all such cases must be regarded as rare exceptions to the usual course which follows the suppuration of the dental nerve.

A nerve may suppurate and a discharge take place through an opening in the crown of a tooth, or from a fang, which may continue for years without occasioning either soreness of the tooth or gum. But if at any future period the outlet of this discharge be stopped, either by plug or pivot, or any other cause, it will most generally find its way through the foramen of the tooth, producing the very same effect as though it had taken place immediately after the suppuration of the nerve.

105, of the American Journal of Dental Science, by incorporating several portions of that paper, into a chapter on the same subject, in his late and very valuable work on the teeth.* As the existence of my essay on that subject may not be remembered by some readers of the Journal, this note will explain the cause of the great similarity of the views of Dr. Robinson and myself on tooth-ache, and particularly on the suppuration of the dental nerve.

* Robinson on the Teeth, page 93.

One of the most prominent pathological conditions of a fang that has been once involved in an abscess of the jaw, where the disease originates in the internal cavity of a tooth, is the existence of that little sac before described, which forms in all such cases at the apex of the fang. It was doubtless this sac that led Mr. Bell into the belief that the cause of abscess was invariably seated within the alveolus. In reference to this he says, "The first effect produced by the irritation of a diseased tooth, or a dead root, is a thickening of the periosteum of the alveolar cavity, which rises the tooth in the socket, and renders it a little loose, and susceptible of considerable pain on pressure; an effusion of coagulable lymph then takes place around the extremity of the fang, which becomes condensed into a sac, in the centre of which pus is formed."* As I have before shown that all the symptoms here referred to by Mr. Bell, as being present in the commencement of abscess, are the result of the suppuration of the dental nerve, and that the little sac which he describes on the extremity of the fang, and attributes to a consolidation of the coagulable lymph, was invariably the result of a discharge from the internal cavity of a tooth; I will therefore only notice, and in a general way the unsoundness of his views, in a pathological sense.

If the first effects of a diseased tooth, or dead root, is a thickening of the periosteum of the alveolar cavity; then the conclusion is imperative, that it is because this fibrous tissue is most easily excited, and, if most easily excited, it is here where the first and greatest amount of inflammation would follow. How then does it happen that coagulable lymph, which is the legitimate effects of inflammation should take place around the extremity of the fang, and at the extremity only, and that no deposite of lymph whatever takes place on the periosteum of the alveolus, where the inflammatory action first commenced; and how does it happen that the coagulable lymph which is effused around the extremity of the fang, should always be found in the same state, whether in the commencement of the disease, or in its latter stage? Be the sacs small or large, it is always the

* Bell on the Teeth, page 216.

same, always fleshy, always condensed. If I mistake not, these interrogations cannot be answered in accordance with any of the known and well established principles of physiology.

In addition to the evidence I have already adduced in support of the doctrine, that the sac which is formed at the extremity of a fang, is invariably caused by a discharge from the internal cavity of a tooth; I will offer as farther proof, the facts set forth in Mr. Bell's description of the peculiar form which these sacs assume; he says, "these sacs assume different appearances, depending upon the nature of the teeth upon which they have been formed. Thus, at the extremity of a round root, the internal cavity of which is simple, the sac is single, and of an oblong or pyriform figure; but in the case of its being formed upon a bicuspid tooth, the root of which is longitudinally contracted, so as to separate the internal cavity into two, having each a distinct foramen at the point, the sac is often double, and has the appearance of two small globular sacs united together."* If the cause of abscess is invariably seated within the alveolus, and the effect of this cause a deposit of coagulable lymph, which is condensed into a sac at the extremity of a fang, why does this peculiar relation of the sac to the internal cavity of a tooth exist? How does it happen, that where there is but one internal cavity to a fang, there is always one sac; but where there are two internal cavities, there are always two sacs? This uniform relation of the sac to the condition of the internal cavity of a fang, proves conclusively, that there is a connection between the two; then if a connection exists, it must be a diseased connection; and I must therefore insist that this diseased connection consists in a discharge from the internal cavity of a fang, explaining at once, the origin and formation of the sac, and the uniform relation which it always holds to each foramen that may exist in a fang.

Prof. Harris in his notice of a paper, that was published some time since, in which I attempted to show that the true abscess of the antrum maxillare, as well as that of the alveoli, originated in a discharge from the internal cavity of a tooth, says, "the pulp,

* Bell on the Teeth, page 216.

or ganglion, as some French writers term it, may suppurate, and the matter be confined in the cavity of the tooth for a long time, without causing alveolar abscess, and the purulent matter contained in the sac at the extremity of the root of a tooth, is not formed, as Mr. Hullihen supposes, in the cavity of the organ. The alveolo-dental membranes at the apex of the root of a tooth around the nerve cord, are more vascular and are endowed with greater nervous sensibility, than at any other part, consequently the inflammatory action here is always the greatest, and it is here suppuration first takes place."*

While I freely admit that Prof. Harris is excellent authority on most subjects of which he treats, yet I must insist that his views in relation to this disease are both inconclusive and problematical. That the nerve may suppurate, and the matter be confined in the cavity of a tooth for a long time without causing abscess, is a fact I admit, and have already explained. To merely proclaim that the matter confined in the sac at the extremity of the root of a tooth is not formed in the cavity of that organ, to say the least, is a very inconclusive kind of refutation. But to contend that the alveolo-dental membranes at the apex of the root of a tooth, are more vascular and are endowed with greater nervous sensibility than any other part, is a doctrine that is new, highly problematical and incongruous. The alveolo-dental membranes situated as they are, between two bones are necessarily endowed with but little vascularity and nervous sensibility, else the comminution of the food could not be endured, is it reasonable then that the apex of the root, the very place of all others most likely to be affected in mastication, should be endowed with greater nervous sensibility than any other part?

The office of the periosteum of the teeth and alveoli have long since been settled, and are well understood. How then does it happen that this greater vascularity and nervous sensibility at the apex of the roots have been overlooked until now, and if overlooked, what are the purposes of such an organization? The fact is, no such an organization exists; it is true, however, that when the periosteum of a tooth becomes inflamed, it is

* Harris' Principles and Practice of Dental Surgery, page 459.

almost invariably at the apex of the fang, occasioned, as I have already shown, by a discharge from the internal cavity of the tooth. It was doubtless owing to this condition of the fang that Prof. Harris has been led to suppose that the periosteum at its extremity was more vascular, and endowed with a greater nervous sensibility than any other place.

But supposing this greater organization of the periosteum at the extremity of a fang could be clearly demonstrated, Professor Harris must doubtless acknowledge that the different kinds of irritation to which the nerve is exposed from the encroachment of dental caries, to be one of the most frequent exciting causes of the inflammation at the extremity of the fang, of which he speaks; and likewise, that the nerve or pulp of a tooth is more highly vascular, and endowed with greater sensibility than the alveolo-dental membranes are at the extremity of the roots; then if the nerve or pulp is most vascular, and greatly the most sensitive, and exposed as it is more directly to irritations of different kinds, is it not most rational to conclude, that inflammation and suppuration would take place in the nerve or pulp before it would occur so remotely from the seat of the exciting cause, as at the extremity of the fang? This conclusion appears to me to be self-evident.

As sacs differ much in size and appearance after an abscess of the jaw has once occurred, it may be well to notice some of the most common features attending this stage of their existence; in all such cases more or less of a rupture may be found to exist in them, sometimes the sac will be so small, that it appears like a thickening of the periosteum only; sometimes they are as large as a pea, well defined, and full of pus, sometimes the rupture in the sac is opposite the foramen of the fang, and very small, sometimes in the side and very large, but in all cases the bone of the fang is completely denuded so far as the sac extends, proving that the matter collects between the periosteum, and the bone of the fang, and that the sac is nothing more than a separation of the periosteum from the bone, and filled with pus from the internal cavity of the tooth as I have already shown. The existence of a sac, therefore, should only be regarded as an effect developed in the progress of an abscess, and not a condition of the first cause of the disease.

An abscess of the jaw cannot possibly originate but once in the same root of a tooth. Yet the sac that forms upon the extremity of a root in such cases often keeps up more or less of a discharge through a fistulous opening in the gum for a long time after the occurrence of an abscess, and likewise renders the gum extremely liable to become inflamed and painful, to swell, and to discharge upon the patient taking the slightest cold. Such attacks of the gum may sometimes occur several times in the course of a month, and sometimes not more than once or twice in a year, and should be regarded as the chronic stage of an abscess, and not as separate and distinct attacks of the disease.

Having now examined the origin, progress and development of abscess in the jaws arising in a discharge from the internal cavity of a tooth, I will next proceed to describe the condition of the same disease when it takes place.

*From mechanical violence within the alveolus.*—This variety of abscess of the jaws, originates from the cracking or splitting of weak fangs in the act of inserting pivot teeth—from driving pivots into fangs so forcibly as to bruise the membrane within the alveolus—from heavy blows upon the teeth—from fractures of the alveolar process, and such like causes where direct violence is done to the periosteum of the fang and alveolus.

The first symptom of the disease is a soreness of the tooth or fang that may be involved. This soreness commences soon after the injury has been inflicted, and gradually increases until the gum and face begin to swell, and then the symptoms and progress of the disease are precisely the same as where it originated from a discharge from the dental cavity, save that in some cases the location of the abscess is more particularly confined to the gum itself.

Thus having briefly pointed out the peculiarities that are usual in abscess of the jaws when occasioned by mechanical injuries, I will pass on to a farther description of the same disease when it originates.

*From a conjoined inflammation of the gum and alveolo-dental membrane.*—This form of abscess invariably originates in the edge of the gum, and is most generally induced by the violent irritation to which this part of the gum is exposed in all cases where it extends partly over a fang, or the crown of a wisdom tooth. The disease is therefore almost exclusively occasioned by roots of teeth, and the wisdom teeth of the lower jaw, but may now and then take place from other causes, and about any tooth in the mouth. The inflammation thus occasioned, gradually extends from the gum to the periosteum of the root of a tooth, and of the alveolus, the gum becomes more and more involved; the inflammation then extends to the neighboring parts, more or less pain or swelling accompanies the attack, and finally the formation of an abscess is the result.

The first indications of the disease is an exquisite tenderness of that part of the gum that may project over a fang, or the crown of a wisdom tooth. When a fang is involved, the inflammation in the gum gradually extends around the fang, and it becomes painful upon the slightest pressure; then the gum begins to swell, which sometimes extends to the face; then a pain of a throbbing description is experienced, and matter forms near the base of the fang and edge of the gum.

But where the wisdom tooth is involved, in addition to all the symptoms just described, the face often becomes enormously swollen, the jaws completely closed, and the pain of the most excruciating character; in all such cases the appearance and developments of the abscess are precisely the same as when it originates in a discharge from the internal cavity of a tooth. Yet the pathological condition of the tooth implicated, points out most clearly the difference in the origin of the disease.

Where the periosteum of the root of a wisdom tooth becomes involved in an inflammation with the edge of the gum, the inflammation travels from the base of the root towards its apex, when, from the suppuration of the dental nerve, the inflammation travels from the apex towards the base. When an abscess originates from the first mentioned cause, the dental nerve is never implicated; when from the latter, it is always destroyed; when the first mentioned disease commences about a fang, the abscess always forms near the edge of the gums; when from a discharge through the foramen of a fang, or from the effects of a sac, the abscess forms opposite the extremity of the fang; thus the difference in the origin of the disease is very palpably indicated.

### *Treatment of Abscess of the Jaws.*

The treatment of this disease may be divided into the preventive, the palliative and the radical.

*The preventive treatment*, where the disease is originating from the suppuration of the dental nerve, consists in trepanning or drilling the tooth as soon as matter is formed; the opening should be large enough to admit the free escape of the discharge, and should be made where the bone is thinnest, between the cavity and the external surface; the matter may then be completely dislodged by probing the cavity with a very small silver sound. In cases where the disease is originating in the stoppage of a chronic discharge from the internal cavity of a tooth, either by a plug, a pivot, or in any other way, the cause of the confinement of the matter should be removed upon the first occurrence of any unpleasant symptoms. When a bicuspid or molar is affected in this way, the plug should never be replaced; but where a cuspidatus or either of the incisors is affected, the internal cavity of the fang should be carefully but thoroughly cauterized with the actual cautery, and then the plug or pivot may be replaced with perfect impunity.

The portion of the cautery that is to be introduced in the fang, should be filed as near as possible into the shape of the cavity: the cautery may then be highly heated, and plunged at the same instant, and as deeply as practicable, into the fang, and then withdrawn the same moment; this should be repeated again and again, until every thing of a nervous, fibrous or fleshy nature has been entirely destroyed. The use of the actual cautery upon the teeth has been strongly reprobated by some dental practitioners, who, either on account of their timidity, or the want of that dexterity which is necessary to its successful application, have been led to condemn its use in no measured terms. Yet the correctness of this practice in such cases, and upon such teeth as just described, still remains to be successfully controverted.

*The preventive treatment*, where abscess is originating from mechanical violence within an alveolus, after the occurrence of some outrage upon a fang, or from fracture of the alveolar process, consists simply in removing the cause of irritation as soon as possible after the injury has been inflicted.

*The preventive treatment*, where the disease is originating from a conjoined inflammation of the gum and alveolo-dental membranes, consists in removing promptly the portion of gum that may extend partly over a fang, or wisdom tooth, to such an extent as will prevent all future irritation from the same cause. General or constitutional treatment is of no avail in this stage of the disease.

The second stage of abscess of the jaws commences at that period when the matter is beginning to be effused between the gum (or the soft parts) and the alveolar process; from this time onward the disease always assumes the very same general characteristics, whether it originates within the internal cavity of a tooth, an alveolus, or from an inflammation of the gum and alveolo-dental membranes, and therefore the very same course of treatment must be observed, without any reference to the origin of the disease.

*The palliative treatment* may be commenced soon after the gum and face begin to swell, although, as a general rule in surgery, it is extremely improper to open an abscess before it begins to point, except in cases where the matter is forming under the periosteum, and then for the necessary preservation of the bone, the abscess should be opened as soon as any matter is formed. It is upon this principle that the early opening of an abscess of the jaws is urged, the matter in such cases, after leaving the alveolus, collects between the periosteum and the external surface of the alveoli, and more or less destruction of this process must inevitably follow. To prevent such disastrous consequences, an abscess of the jaw should be opened as soon as the nucleus can be felt, which can always be done shortly after the gum begins to swell, except in cases where it arises from a wisdom tooth of the lower jaw. In this operation, a very slender knife may be employed, by inserting it in the edge of the tumefaction, where it extends farthest into the gum; and in pushing the knife forward until the point reaches the germ of the abscess, then, in the act of withdrawing the knife, an incision should be made, corresponding with the base of the swelling, and parallel with the jaw, from one-half to three-fourths of an inch in length; after the bleeding subsides to some extent, a thin piece of silk or linen, of nearly the length and breadth of the incision, should be spread out between the edges of the wound, so as to prevent reunion from taking place. The use of pledgets of lint or of lunar caustic, for the purpose of accomplishing the same object, are to be deprecated on account of the unnecessary pain which they always occasion.

The employment of hot poultices, which are so generally and indiscriminately used, alike both as a preventive and a promoter of an abscess, is of doubtful value, except so far as their warmth and moisture may ameliorate the condition of the skin, in cases where it becomes more or less inflamed; and in such cases, cloths wrung out of hot brandy, and applied as warm as the patient can bear, is decidedly preferable. General treatment sometimes may be resorted to, with a view of controlling the constitutional effects that may arise in this stage of the disease, but never with a view of subduing the disease itself.

The third or chronic stage of abscess of the jaws constitutes cases where more or less matter escapes through a fistulous opening, and where the gum is liable to inflame, swell and discharge every time the patient may take a cold. This form of disease may always be speedily relieved by making a free incision down to the bone, through the inflamed portion of the gum. The employment of leeches, to whatever extent, in such cases, is inadequate, because such attacks of the disease result more from an increased collection of matter, than of an inflammatory action.

*The radical treatment* of abscess of the jaws, I need scarcely add, consists in the removal of the tooth, or the roots of a tooth that may be involved in the disease; this is always the most successful and the most rational course of treatment, and should be adopted in all cases where circumstances will admit, and in every case where the exciting cause originates in any way with the wisdom tooth of the lower jaw, so soon as the face begins to swell; for there is no disease of the mouth more painful and unmanageable than abscess when it takes place from this cause.

WHEELING, VA., *Dec.* 10*th*, 1846.

